# Mass spectrometry in medicine: a technology for the future?

**DOI:** 10.4155/fsoa-2017-0053

**Published:** 2017-06-12

**Authors:** Liam M Heaney, Donald JL Jones, Toru Suzuki

**Affiliations:** 1 Department of Cardiovascular Sciences & NIHR Leicester Biomedical Research Centre, University of Leicester, Glenfield Hospital, Leicester, UK; 2 Department of Cancer Studies, University of Leicester, RKCSB, Leicester, UK

**Keywords:** biomarkers, mass spectrometry, personalized medicine

Biological markers, or ‘biomarkers,’ have application in diagnostic, prognostic and therapeutic investigations, with an ever-increasing catalog of endogenous biological entities such as proteins/peptides, lipids and metabolites being used in clinical settings. Uses of biomarkers include diagnostic rule in/out tests (e.g., high-sensitive troponin for acute myocardial infarction [1]), risk stratification/prediction of patient outcome (e.g., natriuretic peptides in cardiovascular diseases [2]) and monitoring of response to administered medicines (e.g., metabolic signatures of pharmacological interventions [3]). The use of biomarkers, and subsequently their success in improving personalized clinical information, relies on the collection, processing and analysis of biological samples which should be done to an established protocol. MS, an analytical technology capable of measurements with high levels of reproducibility, precision and accuracy, has received recent interest in clinical research for its potential to extend current capabilities in biomarker discovery, development and validation [4]. Technological advances are not without pitfalls, with delicate balancing of cost-to-benefit and cross-site reproducibility required to justify more widespread implementation. This editorial looks to highlight the major advantages of MS in the clinical laboratory, explain the major hurdles in place and provide an insight into the potential applications for clinical biomarker analysis.

MS is capable of measuring a wide range of biomarkers from small molecules (<50 Da) to large macromolecular structures (>10,000 Da) across large dynamic ranges, suitably placing its use for clinical biomarker analysis. This analysis is achieved through the application of varied MS techniques that can be modified and tuned to target biomarker(s) of interest. In addition, analysis of multiple sample matrices is possible, with MS techniques extending from the traditional blood (plasma/serum) and urine measurements to include volatile and nonvolatile molecules in saliva [5,6], sweat and skin secretions [7], exhaled breath gases [8] and tissue slices [9]. Hyphenated MS techniques, such as LC– or GC–MS, provide increased utility through the separation of many hundreds of biological molecules, thereby reducing analytical complexity. This separation staggers the entry of molecules into the mass spectrometer, reducing the potential number of molecules present per analytical scan. Importantly, conditions employed for chromatographic separations can be manipulated to optimize biomarker sensitivity and minimize analytical run times, therefore improving sample throughput. Further, the inclusion of stable isotopically labeled internal standards corrects for inter-run variation, maximizing reproducibility and accuracy of biomarker quantitation. Extending from chromatographic-based systems, direct analysis methods such as MALDI–MS can provide additive clinical information. For example, the measurement of molecules present on the surface of a tissue biopsy can delineate tissue characteristics to discriminate between cancerous and noncancerous cells [9].

The US FDA has approved several MS-based methods as *in vitro* diagnostic devices including neonatal screening of inborn errors of metabolism, identification of microbial species and measurements of therapeutic drugs in the circulation [10]. Barriers that have slowed the progression of these methods in a clinical setting have been associated with the ability to control sample collection, preparation and analytical interpretation across instrument vendors and models, operators and hospital sites [10]. These additive sources of variation necessitate extensive testing to be performed to ensure that the reproducibility of an assay is robust for common use and that varying results do not affect the validity of the result, and therefore its effectiveness in clinical decision making. Regulatory issues further add to the complexity of clinical adoption of MS-based assays, and are an ongoing matter that is being addressed by involved parties (e.g., industry, healthcare bodies). The constant technological evolution of MS provides cumulative benefits from an analytical viewpoint (e.g., improvements in sensitivity and selectivity) but adoption may be limited by regulatory constraints.

Further extension of these MS-based clinical tests for more widespread use have also been hindered through the demands required to install, run and maintain the complex analytical systems. High initial costs for equipment purchase and the facilities required to house and operate the instruments places an enormous financial burden on any hospital or healthcare system that wishes to implement MS into its clinical assessments. Although costs remain high, more affordable mass spectrometers have been introduced into the commercial market, albeit often providing reduced functionality to their more expensive counterparts. A major stumbling block for hospital-based MS is the requirement for a constant flow of inert gas(es) and the space required to place both the instrumentation and the background equipment required for operation (e.g., vacuum pumps, air compressors and gas generators). Many of the modern mass spectrometers available exhibit a reduced footprint and can be combined with purpose-built benches that contain the necessary background equipment within a sound-insulated base. Although this arrangement is not preferred, it allows MS use in laboratories, which may not have otherwise suitable facilities. Furthermore, the development of more user-friendly software packages has simplified the training for novice users and subsequently reduced the reliance on highly experienced personnel for routine analytical operation. However, this cannot negate the specific expertise and proficiency required for MS method development and validation and therefore laboratories must be supported by technical experts, increasing operation costs.

Clinical integration of MS is being enhanced through the development of instruments intended for use at the point-of-care (POC). POC devices are vital for rapid identification and characterization of biomarkers, with the modification and miniaturization of MS-based systems driving a real potential for the implementation of MS at the bedside. Leading this innovation is the development of the iKnife to cauterize tissue during surgery, directing fumes of vaporized molecules into a mass spectrometer positioned within the operating theater, allowing *in vivo* identification of healthy and diseased tissue [11,12]. Although the iKnife is present within the operating theater, it requires the use of an advanced mass spectrometer that cannot be relocated between surgeries. Recent developments in miniaturization of mass spectrometers offer the potential for sampling in the field (e.g., in ambulances) and at the bedside, expanding the use of biomarkers at the POC. Researchers at Purdue University have pioneered the development of the ‘mini mass spec’, producing an instrument that can be carried by a handle and used in any location [13]. Additionally, the development of paperspray MS has allowed direct analysis of tissue biopsies by applying a solvent spray and high voltage to a triangular-shaped porous paper at the entrance of the mass spectrometer [14]. Further clinical developments have been made in exhaled breath gas analysis, with a compact quadrupole mass spectrometer capable of real-time measurement of exhaled volatiles at the bedside [8], currently being employed into the multicenter East Midlands Breathomics Pathology Node research program [15]. These advancements provide the potential to rapidly screen biological samples to make near-instant clinical judgements within a hospital setting, with added potential to be deployed into general practitioner surgeries for routine health assessments.

With the growing reliance on personalized medicine, MS has an innate ability to improve clinical decision making through the measurements of biomarkers that may not be detectable by alternative methods. For example, current immunoassays to measure BNP for cardiovascular disease suffer from a lack of specificity to detect the intact BNP molecule, also measuring its truncated forms that are co-existent in the circulation [16]. To this end, we recently reported that a selection of these truncated forms was useful in risk stratification of acute heart failure patients [17], and that understanding the distribution of these different forms requires the use of MS. Furthermore, the impact of the gut microbiome has been implicated in disease development. Notably, TMAO, a gut-derived metabolite, was initially characterized using MS-based nontargeted metabolomics [18] and has since shown to be an independent prognostic predictor in multiple cardiovascular disease states [19,20]. Although TMAO is not currently measured in clinic, the necessary steps to incorporate it into routine clinical analysis are being taken. Further MS-based experiments incorporating global ‘omics based approaches, such as metabolomics and proteomics, are likely to continue to uncover novel disease-specific biomarkers that show associative or direct mechanistic relationships with the onset of conditions and/or future risk of adverse events.

To conclude, the major bottlenecks for implementation of MS-based clinical assays are its costs and, perhaps more importantly, the need for methods to be approved by healthcare regulatory authorities who may be hesitant to support MS-based methods without extensive testing across institutions. To overcome this, further work toward simplified and approved analytical kits, as well as the ability to automate preparation and analysis using methods that can be shared globally to provide cross-validation of care across healthcare systems is required. As MS becomes gradually more commonplace in the clinical laboratory, the true benefits of its use will become rapidly apparent and we expect to see many more clinically applied MS methods published in both scientific literature and on regulatory approval lists. It is not said that MS will replace current low-cost and reliable clinical tests, but as biomarker testing evolves MS has the ability to broaden the inventory of disease-specific molecules and place itself within a clear niche in which to excel.

## References

[1] Thelin J, Melander O, Ohlin B (2015). Early rule-out of acute coronary syndrome using undetectable levels of high sensitivity troponin T. *Eur. Heart J. Acute Cardiovasc. Care*.

[2] Volpe M, Rubattu S, Burnett J (2014). Natriuretic peptides in cardiovascular diseases: current use and perspectives. *Eur. Heart J.*.

[3] Kaddurah-Daouk R, Weinshilboum R (2015). Metabolomic signatures for drug response phenotypes: pharmacometabolomics enables precision medicine. *Clin. Pharmacol. Ther.*.

[4] Jannetto PJ, Fitzgerald RL (2016). Effective use of mass spectrometry in the clinical laboratory. *Clin. Chem.*.

[5] Martin HJ, Riazanskaia S, Thomas CL (2012). Sampling and characterisation of volatile organic compound profiles in human saliva using a polydimethylsiloxane coupon placed within the oral cavity. *Analyst*.

[6] Criado-Garcia L, Ruszkiewicz DM, Eiceman GA, Thomas CL (2016). A rapid and non-invasive method to determine toxic levels of alcohols and gamma-hydroxybutyric acid in saliva samples by gas chromatography-differential mobility spectrometry. *J. Breath Res.*.

[7] Riazanskaia S, Blackburn G, Harker M, Taylor D, Thomas CL (2008). The analytical utility of thermally desorbed polydimethylsilicone membranes for in-vivo sampling of volatile organic compounds in and on human skin. *Analyst*.

[8] Heaney LM, Ruszkiewicz DM, Arthur KL (2016). Real-time monitoring of exhaled volatiles using atmospheric pressure chemical ionization on a compact mass spectrometer. *Bioanalysis*.

[9] Calligaris D, Feldman DR, Norton I (2015). MALDI mass spectrometry imaging analysis of pituitary adenomas for near-real-time tumor delineation. *Proc. Natl Acad. Sci. USA*.

[10] FDA Public Workshop on Liquid Chromatography-Mass Spectrometry (LC-MS) in the Clinic (UCM 496805) (2016). http://www.fda.gov/downloads/MedicalDevices/NewsEvents/WorkshopsConferences/UCM496805.pdf.

[11] Balog J, Sasi-Szabo L, Kinross J (2013). Intraoperative tissue identification using rapid evaporative ionization mass spectrometry. *Sci. Transl. Med.*.

[12] Balog J, Kumar S, Alexander J (2015). *In vivo* endoscopic tissue identification by rapid evaporative ionization mass spectrometry (REIMS). *Angew. Chem. Int. Ed. Engl.*.

[13] Gao L, Song Q, Patterson GE, Cooks RG, Ouyang Z (2006). Handheld rectilinear ion trap mass spectrometer. *Anal. Chem.*.

[14] Wang H, Manicke NE, Yang Q (2011). Direct analysis of biological tissue by paper spray mass spectrometry. *Anal. Chem.*.

[15] The East Midlands Breathomics Pathology Node (2017). http://embernode.org.

[16] Miller WL, Phelps MA, Wood CM (2011). Comparison of mass spectrometry and clinical assay measurements of circulating fragments of B-type natriuretic peptide in patients with chronic heart failure. *Circ. Heart Fail.*.

[17] Suzuki T, Israr MZ, Heaney LM, Takaoka M, Squire IB, Ng LL (2017). Prognostic role of molecular forms of B-type natriuretic peptide in acute heart failure. *Clin. Chem.*.

[18] Wang Z, Klipfell E, Bennett BJ (2011). Gut flora metabolism of phosphatidylcholine promotes cardiovascular disease. *Nature*.

[19] Suzuki T, Heaney LM, Bhandari SS, Jones DJ, Ng LL (2016). Trimethylamine N-oxide and prognosis in acute heart failure. *Heart*.

[20] Suzuki T, Heaney LM, Jones DJ, Ng LL (2017). Trimethylamine N-oxide and risk stratification after acute myocardial infarction. *Clin. Chem.*.

